# Didox and resveratrol sensitize colorectal cancer cells to doxorubicin via activating apoptosis and ameliorating P-glycoprotein activity

**DOI:** 10.1038/srep36855

**Published:** 2016-11-14

**Authors:** Sahar A. Khaleel, Ahmed M. Al-Abd, Azza A. Ali, Ashraf B. Abdel-Naim

**Affiliations:** 1 Department of Pharmacology and Toxicology, Faculty of Pharmacy, Al-Azhar University, Nasr City, Cairo, Egypt; 2 Pharmacology Department, Medical division, National Research Centre, Dokki, Giza, Egypt; 3 Department of Pharmacology and Toxicology, Faculty of Pharmacy, Ain-Shams University, Abbasia, Cairo, Egypt.

## Abstract

Doxorubicin (DOX) has limited efficacy in colorectal cancer due to multi-drug resistance. Resveratrol (RES) and didox (DID) are polyhydroxyphenols with potential chemosensitizing effects. Herein, we assessed the chemomodulatory effects of RES and DID to DOX in colorectal cancer cells. Equitoxic combination of DOX with RES and DID in HCT 116 reduced the IC_50_ of DOX from 0.96 ± 0.02 μM to 0.52 ± 0.05 μM and 0.4 ± 0.06 μM, respectively. Similarly, combination of DOX with RES and DID in HT-29 decreased the IC_50_’s of DOX from 0.88 ± 0.03 μM to 0.47 ± 0.02 μM and 0.29 ± 0.04 μM, respectively. The expressions of p53 and Bax genes were markedly elevated in HCT 116 cells after exposure to DOX/DID. In HT-29 cells, the expression of Bcl-XL gene was significantly decreased after exposure to DOX/DID. In addition, combination of DOX with RES significantly increased the expression of Bax gene in HCT 116 cells. RES treatment induced significant S-phase arrest in DOX-treated HCT 116 cells, while DID induced G_2_/M- and S-phase arrest in HCT 116 and HT-29, respectively. Both RES and DID significantly enhanced the intracellular entrapment of DOX due to blocking the efflux activity of p-glycoprotein pump. In conclusion, RES and DID sensitize colorectal cancer cells to DOX via facilitating apoptosis and enhancing intracellular entrapment of DOX.

Colorectal cancer (CRC) is the third most commonly diagnosed cancer in males and the second in females with an estimated 1.4 million cases and 693.000 deaths occurring in 2012, accounting for 8% of all cancer related deaths^1^. Despite recent advances in chemotherapy, currently used anticancer molecules are unable to improve the prognosis of advanced or recurrent colorectal cancer, which remains incurable^2^.

The anthracycline, doxorubicin (DOX), is a widely used chemotherapy due to its efficacy in fighting wide range of cancers such as carcinomas, sarcomas and hematological neoplasias^3^,^4^. However, there are clinical limitations that arise from its susceptibility to multi-drug resistance^5^. Overexpression of ATP-dependent efflux pump p-glycoprotein (P-gp) and its related proteins is crucial to multidrug resistance (MDR) and chemotherapy failure in cancer treatment^6^. P-gp is encoded by *MDRl* gene; and is considered a member of the ATP-binding cassette (ABC) transporter superfamily. It is energy-dependent transporter pump that removes xenobiotics such as, DOX outward from cells and confer drug resistance in tumor cells^7^,^8^.

Compounds of natural origin are very rich source for leads with potential anticancer properties as well as chemomodulatory effects such as, P-gp inhibitors^7^,^9^,^10^,^11^,^12^,^13^,^14^,^15^. Resveratrol (RES) is naturally occurring plant antibiotic known as phytoalexin, found in various plants, nuts, fruits and especially abundant in grapes and red wine^16^,^17^. It has been extensively studied for its antioxidant, anti-aging and anti-inflammatory activities^18^,^19^,^20^,^21^,^22^,^23^. In addition, *in vivo* and *in vitro* studies showed that RES possesses potential anti-tumor activity against several malignancies^24^,^25^,^26^,^27^. According to our previous study, RES potentiates the cytotoxic properties of DOX in MCF-7, HeLa and HepG2 cells via P-gp inhibition and downregulation of *MDR1* gene^28^.

Didox (DID) is a synthetic polyphenolic compound which shares important biochemical targets with RES^29^. It is potent inhibitor for ribonucleotide reductase enzyme which interferes with DNA synthesis and repair^30^. Ribonucleotide reductase enzyme has been considered potential target for cancer chemotherapy^31^. DID showed anti-tumor effects in a variety of experimental systems, and several human tumor xenografts^32^,^33^,^34^. It may exert its anti-tumor effect via the activation of various apoptosis pathways^35^. According to our previous work as well as other research groups, DID and RES improve the cytotoxic profile of different anticancer agents and protect from their toxic effects^28^,^36^,^37^,^38^,^39^.

DID and RES might be potential successful adjuvant candidates for combination with DOX^33^,^40^. Therefore, we investigated the potential improvement effects of RES and DID on DOX-anticancer properties and the possible underlying mechanisms in two colorectal cancer cell lines with different expression levels of *MDR1* gene.

## Results

### RES and DID improve the cytotoxicity of DOX in colorectal cancer cells

**Figure 1:**
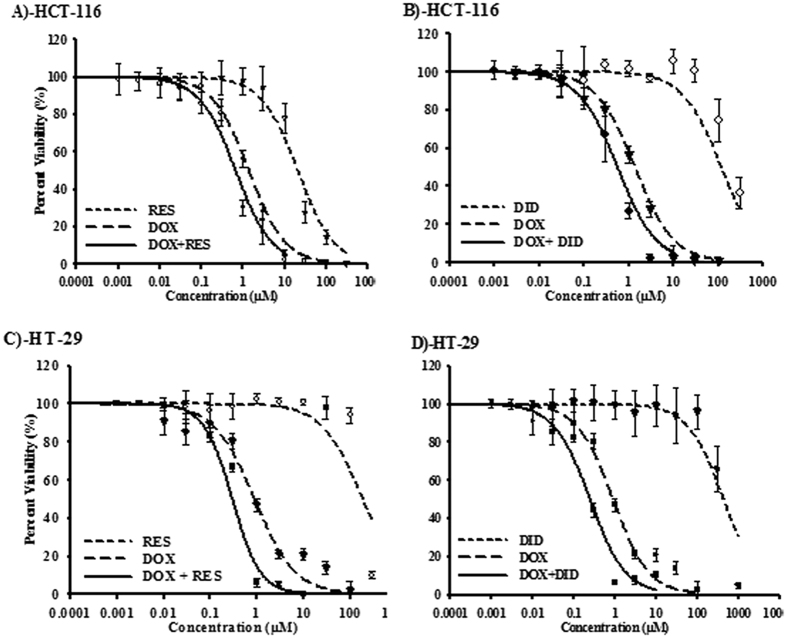
The effect of RES (A&C) and DID (B&D) on the dose response curve of DOX in HCT 116 (A&B) and HT-29 (C&D) colorectal cancer cell line. Cells were exposed to serial dilution of DOX (long dashed lines), RES/DID (short dashed lines) or combination of RES/DID with DOX (solid lines) for 72h. Cell viability was determined using SRB-assay and data are expressed as mean ± SD (n = 3).

To study the effect of RES and DID on the cytotoxic profile of DOX, the dose response curve of DOX alone was assessed relative to its combination with RES or DID in two colorectal cancer cell lines (Fig. 1A–D) (Table 1). In HCT 116 cells, DOX exerted gradient cytotoxic activity with increasing concentration; viability started to drop significantly (P < 0.05) at concentration of 0.3 μM. Cellular log kill was gradual in profile with IC_50_ of 0.96 ± 0.02 μM (Fig. 1A,B). Similarly, RES and DID single treatments exerted gradual cytotoxic activity with increasing concentration; viability started to drop significantly (P < 0.05) at concentrations of 10 μM and 100 μM, respectively. Both RES and DID have steep cellular log kill profile with IC_50_’s of 17.5 ± 02 μM and 105 ± 1.5 μM, respectively (Fig. 1A,B). Equitoxic combination of RES or DID with DOX significantly improved the cytotoxic profile of DOX (Fig. 1A,B). IC_50_’s of DOX after combination with RES and DID were significantly (P < 0.05) decreased from 0.96 ± 0.02 μM to 0.52 ± 0.05 μM and 0.4 ± 0.06 μM, respectively (Table 1). The calculated CI-values for DOX with RES and DID were 1.16 and 0.76, respectively. These CI-values are indicative of additive interaction of DOX with RES and synergistic interaction with DID in HCT 116 cell line (Table 1).

Similarly in HT-29 cell line, DOX exerted gradient cytotoxic activity with increasing concentration; viability started to drop significantly (P < 0.05) at concentration of 0.3 μM (Fig. 1C,D). Cellular log kill was gradual in profile with IC_50_ of 0.88 ± 0.03 μM (Table 1). Also, RES and DID single treatments exerted gradual cytotoxic activity with increasing concentration; viability started to drop significantly (P < 0.05) at concentrations of 100 μM and 300 μM, respectively (Fig. 1C,D). Both RES and DID showed steeping cellular log kill with IC_50_’s of 187.1 ± 4.7 μM and 501.6 ± 53 μM, respectively. Equitoxic combination of RES and DID with DOX significantly improved the cytotoxic profile of DOX in HT-29 cell line (Fig. 1C,D). IC_50_’s of DOX after combination with RES and DID were significantly (P < 0.05) decreased from 0.88 ± 0.03 μM to 0.47 ± 0.02 μM and 0.29 ± 0.04 μM, respectively (Fig.1C,D) (Table 1). The calculated CI-values for DOX with RES and DID were 1.02 and 0.6, respectively. These CI-values are indicative of additive interaction for DOX with RES and synergistic interaction characteristics for DOX with DID in HT-29 cell line (Table 1).

### RES and DID enhance DOX induced apoptosis in colorectal cancer cells

**Figure 2:**
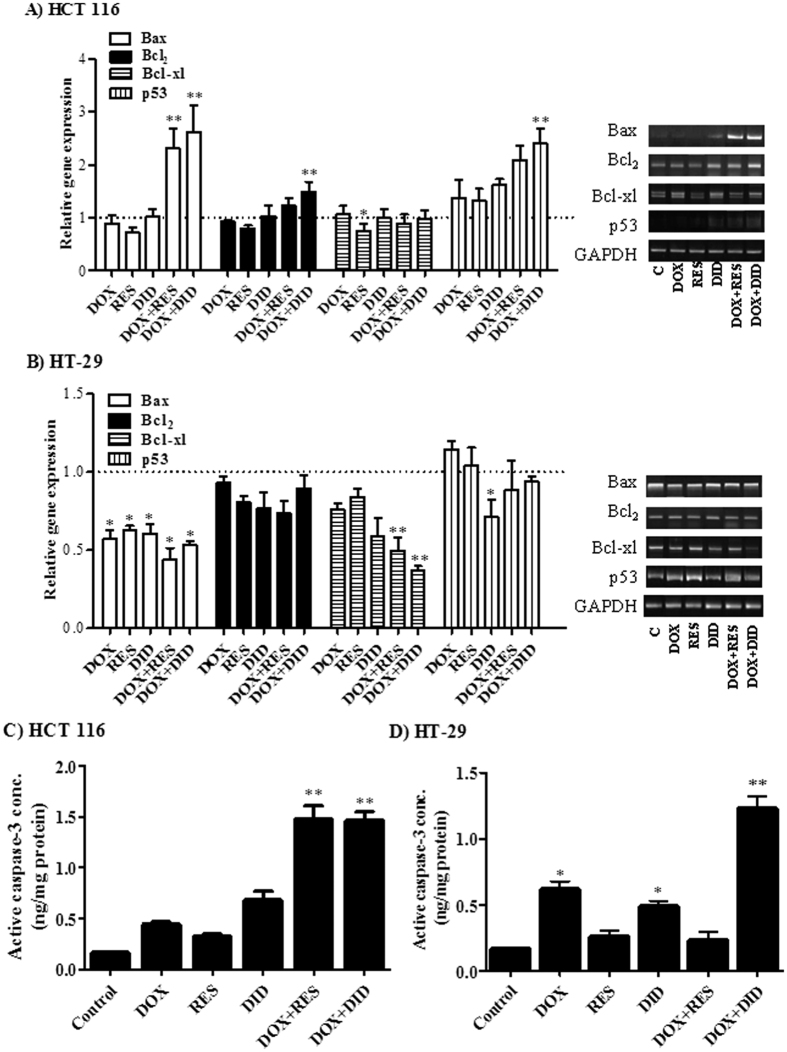
Gene expression of Bax, Bcl_2_, Bcl-xl and p53 was determined in HCT 116 (**A**) and HT-29 (**B**) cells using RT-PCR after treatment with DOX, RES, DID or their combination for 24 h. Concentration of active caspase-3 was assessed in HCT 116 (**C**) and HT-29 (**D**) cells after treatment for 48 h. Data are expressed as mean ± S.D. (n = 3). *Significantly different from control group (p < 0.05). **Significantly different from DOX treatment alone (p < 0.05).

To understand the interaction characteristics of DOX with RES or DID, gene expression analysis for apoptosis key markers was assessed using real time PCR. In DOX-treated HCT 116, the apoptotic gene, Bax, was not significantly changed compared to the untreated cells. However, combination of RES or DID with DOX significantly increased the relative Bax gene expression by two fold compared to DOX-treated cells (Fig. 2A). On the other hand, Bcl_2_ antiapoptotic gene was not significantly changed in DOX treatment compared to the untreated cells. Surprisingly, combination of DID with DOX significantly (p < 0.05) increased the relative Bcl_2_ gene expression (Fig. 2A). Moreover, the expression of the antiapoptotic gene, Bcl-XL, was significantly decreased after treatment with RES alone compared to control cells. All other treatments did not show any significant change in the expression level of Bcl-XL compared to control cells (Fig. 2A). Interestingly, p53 level was significantly over expressed after exposure to combination of DID with DOX (Fig. 2A). This might be attributed to overweighing the total apoptotic signal relative to the anti-apoptotic signal in DOX/DID-treated HCT 116 cells. Accordingly, the effect of RES and DID on the intracellular concentration of active caspase-3 was assessed. In HCT 116, there was no significant change in active caspase-3 concentration in all single treatments. DID and DOX treatment alone induced apparently higher level of caspase-3; however did not reach a significant level (p < 0.05). Yet, combination of RES or DID with DOX increased the concentration of the active form of caspase-3 by 3.6 and 3.5 fold, respectively (Fig. 2C).

In HT-29 cells, the apoptotic gene, Bax, was significantly under expressed in all single and combined treatments (Fig. 2B). On the other hand, Bcl_2_ antiapoptotic gene was not significantly changed in all treatment groups compared to untreated cells (Fig. 2B). Moreover, the apoptotic gene, p53, was significantly under expressed upon single DID treatment. Combination of RES or DID with DOX had no effect on p53 expression (Fig. 2B). Nonetheless, the antiapoptotic gene, Bcl-XL, was not under expressed after single DOX treatment. However, combination of RES or DID with DOX significantly decreased the expression level of Bcl-XL (Fig. 2B). Accordingly, the effect of RES and DID on the intracellular concentration of active caspase-3 was assessed. DOX or DID treatment alone significantly increased the intracellular concentration of active caspase-3 compared to control cells. Yet, combination of DOX/DID further increased the intracellular concentration of active caspase-3 significantly higher than single DOX treatment (Fig. 2D).

### RES, DID, DOX and their combination influence cell cycle distribution of colorectal cancer cells

**Figure 3:**
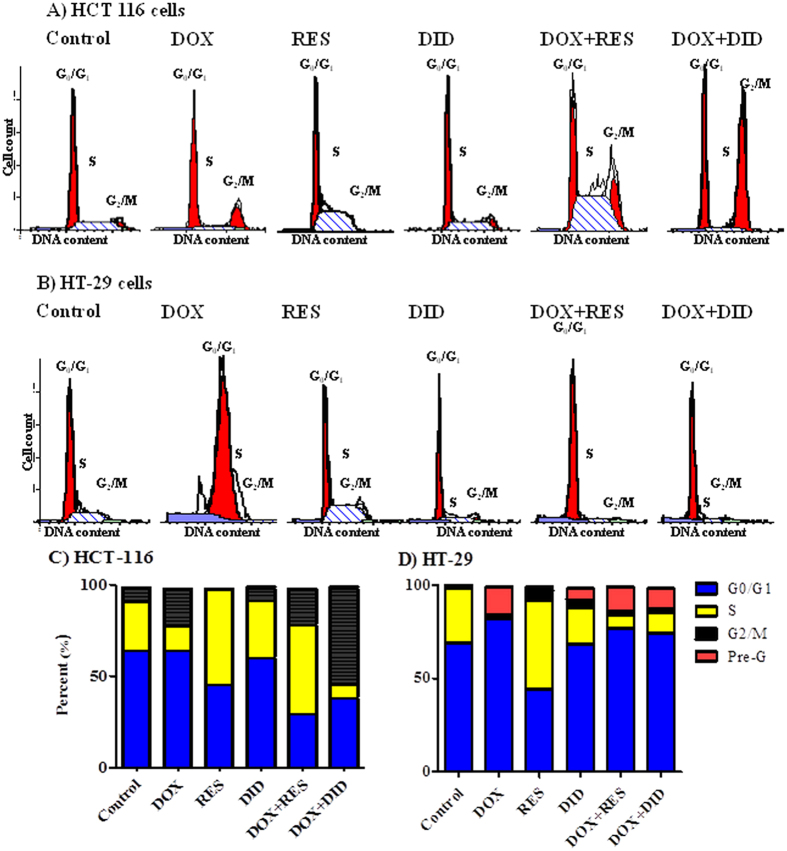
HCT 116 (**A**) and HT-29 (**B**) cells were exposed to DOX, RES, DID and their combinations for 48 h. Cell cycle distribution was determined using DNA cytometry analysis and percentages of cell phases were plotted (**C,D**) compared to control cells; (n = 3).

DNA flow-cytometry was used to assess the effect of RES and DID alone or in combination with DOX on the cell cycle distribution of HCT 116 and HT-29 cell lines. In HCT 116, DOX showed no significant change in cell population in G0/G1-phase compared to the control cells, while both RES and DID decreased cell population in G0/G1-phase from 63.8 ± 0.9% to 45.4 ± 1.3% and 60.5 ± 0.3% respectively. Combination of DOX with RES and DID markedly decreased cell population in G0/G1-phase to 29.5 ± 1.8% and 38.8 ± 1.6% respectively compared to DOX alone (64.95 ± 0.4%) (Fig. 3A,C). In addition, DOX alone decreased cell population in S-phase from 27.8 ± 1.1% to 13 ± 0.8%. On the other hand, both RES and DID single treatments increased the S-phase population to 52.9 ± 1.2% and 31.0 ± 0.4% respectively (control cells in S-phase were 27.8 ± 0.9%). Combination of DOX with RES caused a marked increase in S-phase population to 49.6 ± 1.7%. on the other hand, DOX combination with DID showed significant decrease of S-phase population to 7.0 ± 0.7% compared to DOX treatment alone (13 ± 0.8%) (Fig. 3A,C). DOX treatment showed significant increase in G2/M-phase population from 7.4 ± 0.7% to 20.6 ± 0.7%. RES showed marked decrease in G2/M-phase cell population to 0.8 ± 0.1%. On the other hand, no marked change in G2/M-phase population was induced by treatment with DID compared to control cells. Co-treatment of DOX and RES had no significant change in the G2/M-phase population compared to DOX alone, while combination of DOX with DID induced a significant increase in the G2/M-phase (53.0 ± 3.1%) compared to DOX treatment alone (20.6 ± 0.7%) (Fig. 3A,C).

In HT-29, DOX significantly increased cells in G0/G1-phase from 69.6 ± 1.0% to 82.3 ± 1.0%, while RES significantly decreased cell population in G0/G1-phase to 44.3 ± 0.7%. DID did not induce any significant change in G0/G1-phase cell population. Combination of DOX with RES and DID significantly decreased G0/G1 cell population to 77.0 ± 2.1% and 74.2 ± 0.4%, respectively compared to DOX treatment alone (82.3 ± 1.0%) (Fig. 3B,D). DOX alone abolished cells in S-phase from 29.4 ± 1.1% to 1.9 ± 0.5%. RES induced significant increase in S-phase cells from 29.4 ± 0.6% to 48.1 ± 0.8% compared to control cells. DID moderately decreased S-phase population to 20 ± 1.0%. Combination of DOX with RES or DID increased S-phase population from 1.9 ± 0.8% to 7.6 ± 0.5% and 11.4 ± 1.3%, respectively compared to DOX treatment alone (Fig. 3B,D). No significant change in cell population in G2/M-phase upon DOX treatment. However, significant increase in G2/M phase was noticed by RES (5.2 ± 0.7%), and DID (4.4 ± 0.6%) compared to control cells (0.1 ± 0.2%). Treatment with DOX, RES or DID significantly increased the apoptotic Pre-G phase from 0.9 ± 0.1% to 15.5 ± 1.5%, 2.4 ± 0.1 and 6.7 ± 0.5%, respectively. Combination of DOX with DID or RES did not induced further increase in the apoptotic Pre-G phase population compared to DOX treatment alone (Fig. 3B,D).

### RES and DID increase cellular entrapment of DOX within colorectal cancer cells via inhibiting P-gp efflux pump activity without affecting *MDR1* gene expression

**Figure 4:**
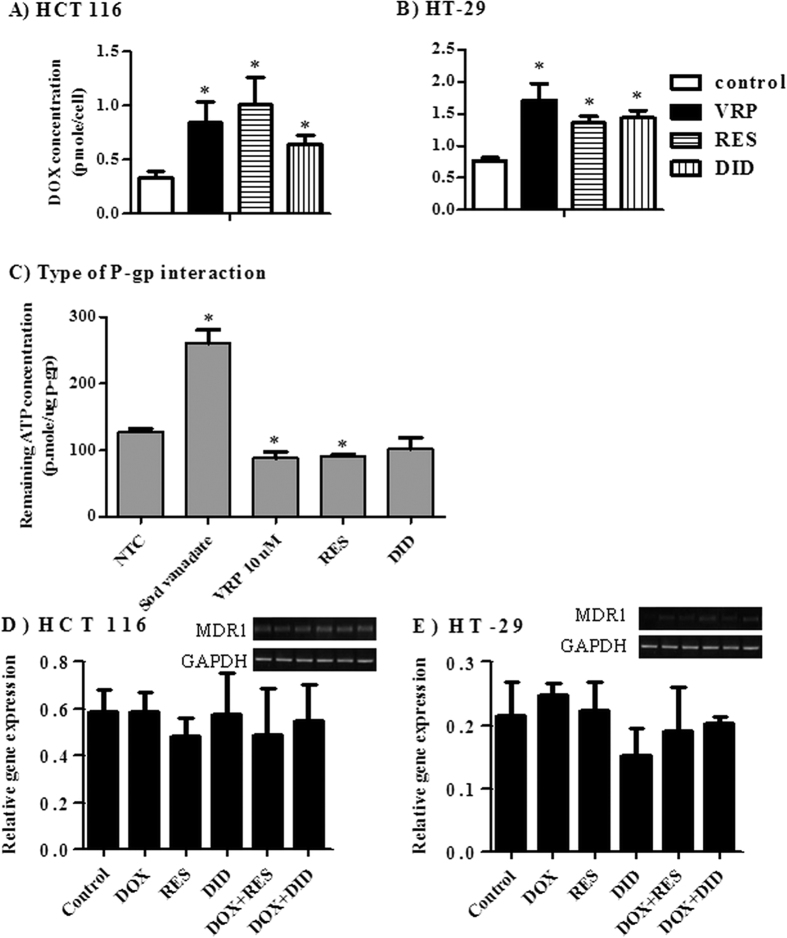
The influence of RES and DID on the intracellular entrapment of DOX was assessed in HCT 116 (**A**) and HT-29 (**B**) cells and compared to positive control agent (VRP). The type of interaction between RES and DID with human recombinant P-gp molecules attached to ATPase subunit was assessed using ATP consumption assay in comparison to VRP and Sod vanadate positive control agents (**C**). Gene expression of MDR1 was assessed in HCT 116 (**D**) and HT-29 (**E**) cells using RT-PCR after treatment with DOX, RES, DID or their combination for 24 h. Data are expressed as mean ± S.D. (n = 3). *Significantly different from control group (p < 0.05).

P-glycoprotein (P-gp) is a plasma membrane efflux pump coded by *MDR1* gene and highly abundant in colorectal cancer cells (*MDR1* gene is highly expressed in HCT 116 cells and weakly expressed in HT-29 cells^41^). Attributed to its fluorescent properties, the intracellular concentration of DOX was determined after incubation with HCT 116 and HT-29 cells in the presence and absence of RES, DID and VRP (positive P-gp inhibitor). RES significantly increased the intracellular concentration of DOX by 202.1% and 68.8% in HCT 116 and HT-29 cells, respectively (Fig. 4A,B). Similarly, DID significantly increased the intracellular concentration of DOX by 94.8% and 85% in HCT 116 and HT-29 cells, respectively (Fig. 4A,B). The positive control P-gp blocker, VRP, resulted in increasing the intracellular concentration of DOX by 154.2% and 120% in HCT 116 and HT-29 cells, respectively (Fig. 4A,B). Inhibition of P-pgp activity might be mediated by inactivating P-gp related ATPase enzyme activity or via competitive covalent binding to P-gp molecules.

To further investigate the sub-molecular interaction between RES and DID with P-gp molecules, ATP consumption assay was carried out on purified human recombinant P-gp molecules attached to ATPase enzyme. VRP (covalent P-gp inhibitor) and sodium vanadate (ATPase inhibitor) were used as positive controls; and are supposed to increase and decrease ATP consumptions, respectively compared to non-treated control (NTC). RES significantly decreased remaining ATP molecules by 29% compared to 31.5% for VRP (Fig. 4C). Interestingly, DID did not show any significant change in ATP consumption (Fig. 4C) compared to NTC.

Finally, the effect of RES and DID on the expression level of *MDR1* gene was measured by real time PCR. In both HCT 116 and HT-29, none of the treatments under investigation showed any significant change in *MDR1* gene expression (Fig. 4D,E). Together with the previous results (Fig. 4A–C), the ability of DID to enhance the intracellular entrapment of DOX within HCT 116 and HT-29 cells could be attributed to dual interaction with P-gp efflux pump via ATPase inhibition and covalent binding.

## Discussion

The activity of P-glycoprotein (P-gp) is involved in multidrug resistance of CRC^42^,^43^. Doxorubicin (DOX) is a chemotherapeutic drug which has been widely used over the past four decades for the treatment of several humoral and solid neoplasias^4^. DOX might not be the optimum clinical choice for CRC which might be attributed to intrinsic resistance to DOX^44^. In this study, we explored the potential influence of RES and DID on the cytotoxic profile of DOX in two different CRC cells (HCT 116 and HT-29) and its associated mechanism of action/resistance.

According to our data, DID *per se* did not show any promising cytotoxicity in CRC cells under investigation (HCT 116 and HT-29). In the current work as well as in our previous publication, RES showed weak cytotoxicity against HCT 116 cells (IC_50_ 17.5 μM)^28^. At low concentrations, RES decreased DOX-induced cytotoxicity in HCT 116. While at higher concentrations (above 2 μM), RES enhanced DOX-induced viability inhibition in a concentration dependent manner (Fig. 1). This finding is consistent with Chen *et al*.^45^ who demonstrated an opposing effect of RES at low vs. high concentrations. In the present study, RES showed additive while DID showed synergistic effect with DOX against HCT 116 and HT-29 cell lines (Table 1). Similarly, we showed synergistic interaction between RES and DOX or docetaxel in MCF-7 cell line and additive interactions against HeLa and HepG2 cells^28^. In addition, the combination of RES or DID with transtuzumab resulted in synergistic effect in MCF-7 and T47D cells^36^. Moreover, the combination of DID with DOX showed additive interaction in liver cancer cells^39^.

The effects of combining RES or DID with DOX on some apoptosis key markers (Bax, Bcl_2_, p53 and Bcl-XL) were further investigated. According to our observations, in HCT 116 cell line, RES enhanced DOX-induced Bax gene expression which might explain the significant increase in active caspase-3^46^. On the other hand, DID significantly enhanced DOX-induced Bcl_2_, Bax and p53 gene expression. Yet, p53 promotes apoptosis through down-regulating the antiapoptotic Bcl_2_ and up-regulating the apoptotic Bax protein expression at transcriptional level^47^. Thus, p53 might be increased in attempt to down regulate the elevated level of Bcl_2_ gene expression (Fig. 2A,B). Moreover, the significant increase in p53 expression might explain the G_2_/M cell cycle arrest induced in DID/DOX-treated group^48^. This is consistent with the study of Zhang and coworkers who found that genistein, another polyphenolic compound, induces G_2_/M cell cycle arrest and apoptosis in p53-dependent manner^49^. Again, p53 can exert a direct pro-apoptotic function within mitochondrial pathway of apoptosis which is known as the circuitry of p53 death signaling^50^. In another study, DID resulted in DNA damage and p53 induction resulting in apoptosis for acute myeloid leukemia cells^51^. Furthermore, the over expression of p53 and Bax in DID/DOX-treated group might interpret the significant increase in active caspase-3 in HCT 116^46^,^52^. As Bcl-XL overexpression resulted in inhibition of caspase-3 activity^53^, in HT-29 cell line, the increase in caspase-3 activity in DID/DOX treated group might be attributed to the reduction in Bcl-XL expression.

DID was suggested previously to induce apoptosis in other cancer cell lines such as, multiple myeloma cells^33^. Moreover, DID synergizes the anti-tumor effect of temozolomide^54^, carmustine^40^ and cidofovir^34^ against malignant brain tumor cells, DAOY human medulloblastoma cells and Epstein-Barr virus (EBV)-positive nasopharyngeal carcinoma xenografts, respectively. DOX-induced cytotoxicity is partly attributed to the generation of reactive oxygen species and free radicals^55^. DID possesses robust antioxidant activity and potent free radical scavenging capability^56^. Despite possessing potent antioxidant properties, DID did not antagonize DOX-induced cytotoxicity herein as well as in our previous work against liver cancer cells^39^. On contrary, DID can be described to possess sensitizing role for DOX-induced cytotoxicity in CRC cells. This might be similar to the effect of trimidox, another ribonuclueotide reductase inhibitor, which exhibits both anticancer and potent antioxidant properties^57^. DID is known to be a ribonucleotide reductase enzyme inhibitor which might partly explain its anti-proliferative and chemosensitizing effects^33^. Similar to DID, RES has an antioxidant effect^58^ which did not prevent its potentiating effect to DOX-induced cytotoxicity in more than solid tumor type^28^. Many mechanisms have been suggested for RES induced cytotoxicity; yet, the exact mode of action is still controversial^59^.

Further illustration to the potential underlying chemosensitizing effects of RES and DID on DOX might be taken from cell cycle analysis. DOX induced cytotoxicity is suggested to be cell cycle dependent^60^. RES blocked the cell cycle progression in the S-phase in both cell lines which is also consistent with previous studies^61^. In HCT 116, RES showed very strong S-phase arrest in DOX-treated cells, while in HT-29 it exerts mild S-phase arrest compared with DOX-alone (Fig. 3). S-phase arrest generates higher cell population sensitive to S-phase specific agents such as DOX and would be partly the reason for potentiating DOX-induced cytotoxicity^28^. On the other hand, DID induced G_2_/M cell cycle arrest in DOX-treated HCT 116 cells which might add more evidence to the sensitizing effect of DID to DOX-induced cytotoxicity (Fig. 3). Mittal and coworkers demonstrated that the sensitizing effect of berberine to DOX-induced cytotoxicity was associated with a G_2_/M-phase arrest^62^. Moreover, DID/DOX combination induced significant increase in S-phase with reciprocal decrease in G_0_/G_1_-phase compared to DOX treatment alone in HT-29 (Fig. 3). S-phase arrest induced by the combination of DID with DOX may add an extra evidence to the sensitizing effect of DID to DOX in HT-29 cells^63^. In support of the importance of cell cycle arrest to DOX cytotoxicity, Ling and colleagues^60^ found that P388 cells synchronized in S and G_2_/M phases were more sensitive to DOX than cells in G_1_-phase. Strategies to increase G_2_/M arrest have also been associated with enhanced apoptosis^64^.

Although no effect was observed for either DID or RES on the expression level of *MDR1* gene, both agents significantly decreased the P-gp pumping activity (Fig. 4). In our previous work and herein, we showed that RES via covalent binding inhibits P-gp activity in different solid tumor cell lines^28^. To the best of our knowledge, this is the first time to prove P-gp-inhibitory effect attributed to DID via dual P-gp covalent binding and ATPase inhibition. This may shed the light to a field for using DID in improving cellular pharmacokinetics of different antitumor drugs via increasing their intracellular concentrations thus enhancing their cell killing effect.

In conclusion, DID showed superior improvement to DOX-induced cytotoxicity in CRC cells compared to RES. This potentiating effect might be attributed to enhancing apoptosis, inducing cell cycle arrest and enhancing the cellular pharmacokinetics via P-gp inhibitory activity.

## Materials and Methods

### Chemicals and Drugs

Didox (DID) was generously gifted from Professor Howard L. Elford, Molecules for Health Inc., Richmond, VA, USA. Doxorubicin (DOX), resveratrol (RES), and sulpharodamine (SRB) were purchased from Sigma Chemical Co. (St. Louis, MO, USA). RPMI-1640 media, fetal bovine serum and other cell culture materials were purchased from Lonza Group Ltd. (Basel, Switzerland). Other reagents were of the highest analytical grade.

### Cell culture

Human colorectal cell lines, HCT 116 (ATCC^®^CCL-247) and HT-29 (ATCC^®^HTB-38), were obtained from the Vaccera (Giza, Egypt). Cells were maintained in RPMI-1640 supplemented with streptomycin (100 μg/mL); penicillin (100 units/mL) and heat-inactivated fetal bovine serum (10% v/v) in a humidified, 5% (v/v) CO_2_ atmosphere at 37 °C.

### Cytotoxicity assays

The cytotoxicity of DOX, RES and DID were tested against HCT 116 and HT-29 cells by SRB assay as previously described^65^. Exponentially growing cells were collected using 0.25% Trypsin-EDTA and plated in 96-well plates at 1000–2000 cells/well. Cells were exposed to serial concentration of DOX, RES and DID for 72 h and subsequently fixed with TCA (10% w/v) for 1 h at 4 °C. After several washings, cells were exposed to 0.4% (w/v) SRB solution for 10 min in dark place and subsequently washed with 1% (v/v) glacial acetic acid. After drying overnight, Tris-HCl (50 mM, pH 7.4) was used to dissolve the SRB-stained cells and color intensity was measured at λ_max_ of 540 nm and calculated as percent viability of control cells (cells exposed to drug free media)^65^.

### Data analysis

The dose response curves of compounds were analyzed using 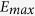 model (Eq. 1).


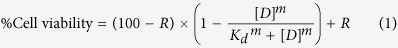


where R is the residual unaffected fraction (the resistance fraction); [D] is the drug concentration used; K_d_ is the drug concentration that produces 50% reduction of the maximum inhibition rate and m is a Hill-type coefficient. IC_50_ is defined as the drug concentration required to reduce absorbance to 50% of that of the control (i.e., K_d_ = IC_50_ when R = 0 and E_max_ = 100 − R)^14^.

Combination index (CI) was calculated as previously described^66^. Briefly, exponentially growing cells were exposed to equitoxic concentrations of RES and DOX or DID and DOX in 96-well plates for 72 h and subsequently subjected to SRB assay. CI was calculated from the formula:


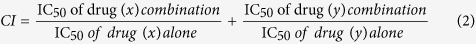


The nature of drug interaction is defined as synergism if CI > 0.8; antagonism if CI < 1.2; and additive if CI ranges from 0.8–1.2.

### RNA extraction, Real time PCR analysis and quantification of gene expression

To assess the gene expression of Bcl_2_, Bax, Bcl-XL, p53 and *MDR1* following treatment of cells with DOX, RES, DID and their combination, total RNA extraction from cells was performed using RNeasy Mini Kit^®^ (Qiagen Inc. Valencia, CA, USA). Reverse transcription was undertaken to construct cDNA library from different treatments using High-capacity cDNA Reverse Transcription Kit (Applied Biosystems, Foster City, CA, USA). The archived cDNA libraries were then subjected to quantitative real time PCR reactions using cyber green fluorophore (Fermentas Inc., Glen Burnie, MD, USA). Primer sequences were as follow: Bcl_2_ forward primer GGG-TAC-GAT-AAC-CGG-GAG-AT and reverse primer CTG-AAG-AGC-TCC-TCC-ACC-AC; Bax forward primer TCT-GAC-GGC-AAC-TTCAAC-TG and reverse primer TGG-GTG-TCC-CAA-AGT-AGG-AG; Bcl-XL forward primer GGC GGA TTT GAA TCT CTT TCT C and reverse primer TTA TAA TAG GGA TGG GCT CAA CC; p53 forward primer CCT-CAC-CAT-CAT-CAC-ACT-GG and reverse primer CTG-AGT-CAGGCC-CTT-CTG-TC; *MDR1* forward primer GCT-GGG-AAG-ATC-GCT-ACT-GA and reverse primer GGT-ACC-TGC-AAA-CTC-TGA-GCA. GAPDH was used as reference housekeeping gene with forward primer TGC-ACC-ACC-AAC-TGC-TTAG and reverse primer GAT-GCA-GGG-ATG-ATG-TTC^67^. *mdr1* Gene expression was normalized using the expression level of corresponding GAPDH gene. The rest of gene expressions in different treatments (single or combined) were expressed relative to its corresponding normalized level in control untreated cells (cells exposed to drug free media) and control treatment gene expression level was expressed as dotted line.

### Assessment of active caspase-3 concentration in colorectal cell lines

To assess the effect of RES, DID, DOX and their combination on apoptosis, the active caspase-3 level was measured by using Quantikine^®^ caspase-3 ELISA kit (R&D Systems, Inc. USA). Briefly, the cells were exposed to the predetermined IC_50_’s of test compounds (single or combined treatments) or drug free media (control group) for 24 h. Cells were harvested and washed with PBS, then incubated with the biotin-ZVKD-fmk inhibitor for 1 hour. Cells were transferred into the wells of a microplate pre-coated with a monoclonal antibody specific for caspase-3. Following a wash to remove any unbound substances, streptavidin conjugated to horseradish peroxidase was added to the wells and bound to the biotin on the inhibitor. Following a wash to remove any unbound Streptavidin-HRP, a substrate solution was added to the wells. The enzyme reaction yields a blue product that turned yellow when a Stop Solution was added. The optical density of each well was determined within 30 minutes, using a microplate reader set to 450 nm with a wavelength correction at 540 nm or 570 nm. The concentrations of active caspase-3 were calculated from a standard curve of constructed with known concentrations of active caspase-3. Caspase concentration was expressed as ng/mg protein. Proteins were determined spectrophotometrically by the method of Bradford using purified bovine serum albumin as a standard.

### Analysis of cell cycle distribution

To determine the effect of DOX, RES, DID and their combinations on the cell cycle distribution in HCT 116 and HT-29 cell lines; cells were exposed to the predetermined IC_50_’s of test compounds (single or combination) for 24 h and compared to control cells (treated with drug free media). Cell cycle analysis was performed using the CycleTEST™ PLUS DNA Reagent Kit (Becton Dickinson Immunocytometry Systems, San Jose, California, USA). Control cells with known DNA content (PBMCs) were used as a reference point for determining the DI (DNA Index) for the test samples. Cells were stained with propodium iodide following the procedure provided by the kit and then run on the DNA cytometer. Cell cycle distribution was calculated using CELLQUEST software (Becton Dickinson Immunocytometry Systems, San Jose, California, USA).

### Assessment of Pgp activity

To assess the effect of RES and DID on the efflux pumping activity of P-gp in tumor cell lines, the P-gp substrate, DOX, was used as a probe and verapamil (VRP) was used as standard p-gp blocker. Briefly, exponentially growing cells were plated in 6-well plates in plating density of 10^5^ cells/well. Cells were exposed to DOX (10 μM), DOX (10 μM) and RES (10 μM), DOX (10 μM) and DID (10 μM) or DOX (10 μM) and VRP (10 μM) for 2 h at 37 °C and subsequently extracellular DOX containing media was washed trice with ice cold PBS. Intracellular DOX were extracted after cell lysis by incubation with SDS (2% w/v), saturated aqueous solution of ZnSO_4_ (100 μl), Acetonitril (500 μl) and acetone (500 μl) for 30 min at 37 °C. After centrifugation, supernatant was collected and DOX concentration was measured spectroflourometrically at λ_ex/em_ 482/550 nm^28^,^68^.

### Assessment of Pgp- ATPase activity

To reveal the mechanism of P-gp-inhibition induced by RES and DID, assessment of P-gp attached ATPase activity was performed using Pgp-Glo™ Assay Systems (Promega Corporation, Madison, Wisconsin, USA). Briefly, in 96-well plates, Pgp-Glo™ Assay Buffer was mixed with Na_3_VO_4_, VRP, RES, or DID. Recombinant human P-gp membrane fraction was incubated with the reaction mixture at 37 °C for about 5 minutes, and then the reaction was initiated by adding 10 μl of 25 mM MgATP and incubating for 40 minutes at 37 °C. An identical reaction mixture without drug treatment (NTC) was assayed in parallel. The reaction was stopped and the remaining un-consumed ATP was detected using luciferase firefly luminescent signal and ATP standard curve was plotted. The remaining concentrations of ATP were expressed as (p. mole/μg P-gp molecules). Sodium vanadate (Na_3_VO_4_) was used as selective inhibitor of P-gp related ATPase enzyme, and samples treated with Na_3_VO_4_ have greater luminescent signal than un-treated samples (NTC). In addition, VRP inhibits pgp molecules via covalent binding and competing with other substrates, hence, stimulating ATPase activity resulting in more consumption of ATP molecules compared to NTC.

### Statistical analysis

Data are presented as mean ± SD. Analysis of variance (ANOVA) with LSD post hoc test was used for testing the significance using SPSS^®^ for windows, version 17.0.0. p < 0.05 was taken as a cut off value for significance.

## Additional Information

**How to cite this article**: Khaleel, S. A. *et al*. Didox and resveratrol sensitize colorectal cancer cells to doxorubicin via activating apoptosis and ameliorating P-glycoprotein activity. *Sci. Rep.* **6**, 36855; doi: 10.1038/srep36855 (2016).

**Publisher’s note:** Springer Nature remains neutral with regard to jurisdictional claims in published maps and institutional affiliations.

## Figures and Tables

**Table 1:** Effect of RES and DID on the cytotoxicity of DOX in HCT 116 and HT-29 cell lines.

	IC_50_ against HCT 116 cells (*μM*)	IC_50_ against HT-29 cells (*μM*)
**DOX**	0.96 ± 0.02	0.88 ± 0.03
**RES**	17.5 ± 02	187.1 ± 4.7
**DID**	105 ± 1.5	501.6 ± 53
**DOX + RES**	0.52* ± 0.05	0.47* ± 0.02
**DOX + DID**	0.4* ± 0.06	0.29* ± 0.04
**CI-value (DOX + RES)**	**Additive/1.16**	**Additive/1.02**
**CI-value (DOX + DID)**	**Synergism/0.76**	**Synergism/0.6**

*Significantly different from DOX alone at p-value < 0.05.
